# Disease-Modifying Treatments and Their Future in Alzheimer’s Disease Management

**DOI:** 10.7759/cureus.56105

**Published:** 2024-03-13

**Authors:** Blake Smith, Raymond L Ownby

**Affiliations:** 1 Psychiatry and Behavioral Sciences, Nova Southeastern University Dr. Kiran C. Patel College of Osteopathic Medicine, Davie, USA

**Keywords:** anti-amyloid, neurodegenerative disesase, neuro inflammation, anti-tau therapies, alzheimers disease

## Abstract

Alzheimer’s disease (AD) is a progressive neurodegenerative disorder characterized by memory impairment, a loss of cholinergic neurons, and cognitive decline that insidiously progresses to dementia. The pathoetiology of AD is complex, as genetic predisposition, age, inflammation, oxidative stress, and dysregulated proteostasis all contribute to its development and progression. The histological hallmarks of AD are the formation and accumulation of amyloid-β plaques and interfibrillar tau tangles within the central nervous system. These histological hallmarks trigger neuroinflammation and disrupt the physiological structure and functioning of neurons, leading to cognitive dysfunction. Most treatments currently available for AD focus only on symptomatic relief. Disease-modifying treatments (DMTs) that target the biology of the disease in hopes of slowing or reversing disease progression are desperately needed. This narrative review investigates novel DMTs and their therapeutic targets that are either in phase three of development or have been recently approved by the U.S. Food and Drug Administration (FDA). The target areas of some of these novel DMTs consist of combatting amyloid or tau accumulation, oxidative stress, neuroinflammation, and dysregulated proteostasis, metabolism, or circadian rhythm. Neuroprotection and neuroplasticity promotion were also key target areas. DMT therapeutic target diversity may permit improved therapeutic responses in certain subpopulations of AD, particularly if the therapeutic target of the DMT being administered aligns with the subpopulation’s most prominent pathological findings. Clinicians should be cognizant of how these novel drugs differ in therapeutic targets, as this knowledge may potentially enhance the level of care they can provide to AD patients in the future.

## Introduction and background

Alzheimer's disease (AD) is a neurodegenerative disorder that commonly presents with short-term memory loss that insidiously progresses to dementia. Cognitive decline with a strong association with the loss of cholinergic neurons, impaired memory, and possible motor abnormalities that affect speech, behavior, and visuospatial orientation are characteristic of AD [1]. Unfortunately, this debilitating disease is extremely prevalent. Its prevalence is so widespread that it accounts for up to 60%-80% of all cases of dementia, making it the leading cause [1]. The etiology of AD is multifactorial, but advanced age is the strongest risk factor. This is exemplified by the 10% prevalence rate in individuals over the age of 65 which significantly increases to 40% for those over the age of 80 [1]. The prevalence rate of AD will continue to rise as the growing population of the United States continues to age. Fueled by impressive advancements in life expectancy, the global population aged 65 or older is anticipated to grow from approximately 524 million in 2010 to nearly 1.5 billion by 2050, with the majority of this growth occurring in developing nations [2]. AD will create even more of a burden on health care in the future than it does today. The World Health Organization update on the epidemiology of AD in 2013 predicted the number of people worldwide suffering from dementia (35.6 million) to triple by the year 2050 [3]. This concerning rise in prevalence can be attributed to AD's association with advanced age and an increasing global life expectancy. Despite the frightening prevalence of AD, only symptomatic treatment was largely available for patients until recently. Symptomatic relief of memory impairment and the restored ability to perform some activities of daily living have shown promising results with the use of cholinesterase and N-methyl-d-aspartate inhibitors in patients with AD [4]. The development and introduction of novel treatments that not only relieve symptoms but also modify disease progression are of great importance. 

The pathophysiology of AD is complex. Genetic predisposition, gliosis, neural inflammation, dysregulated production and removal of reactive oxygen species (ROS), mitochondrial dysfunction, disrupted sleep parameters, brain hypometabolism, and excess metal ion accumulation have all been implicated in the pathogenesis of AD [1,5]. This complexity makes the development of efficacious, disease-modifying drugs so difficult.

**Figure 1:**
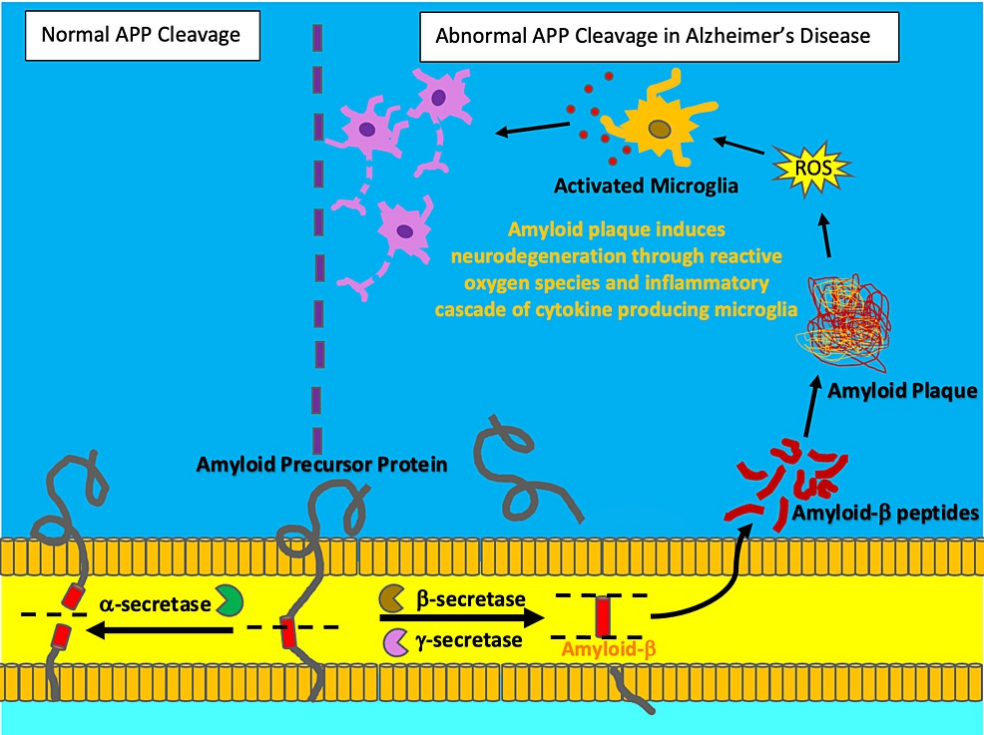
Amyloid plaque formation Under normal conditions, Amyloid precursor protein (APP) is predominantly cleaved by the enzyme, $\alpha$-secretase. However, in Alzheimer's disease, an abnormal increase in the enzymatic activity of $\beta$-secretase and $\gamma$-secretase leads to the misfolding and aggregation of APP proteolytic fragments. These fragments aggregate to form plaques, which subsequently trigger inflammation through oxidative damage from reactive oxygen species (ROS) and the activation of cytokine producing microglia. Chronic neuroinflammation leads to neurodegeneration. The illustration was originally created by the authors.

The histopathological hallmarks found in AD are the formation and accumulation of extracellular amyloid-β plaques and intracellular neurofibrillary tau tangles. Amyloid-β peptides are the proteolytic fragments of the transmembrane receptor, Amyloid Precursor Protein (APP). As illustrated by Figure 1, an abnormal increase in the enzymatic cleavage of APP by β-secretase in AD results in a misfolded amyloid-β protein that is more susceptible to aggregation extracellularly [5].

**Figure 2:**
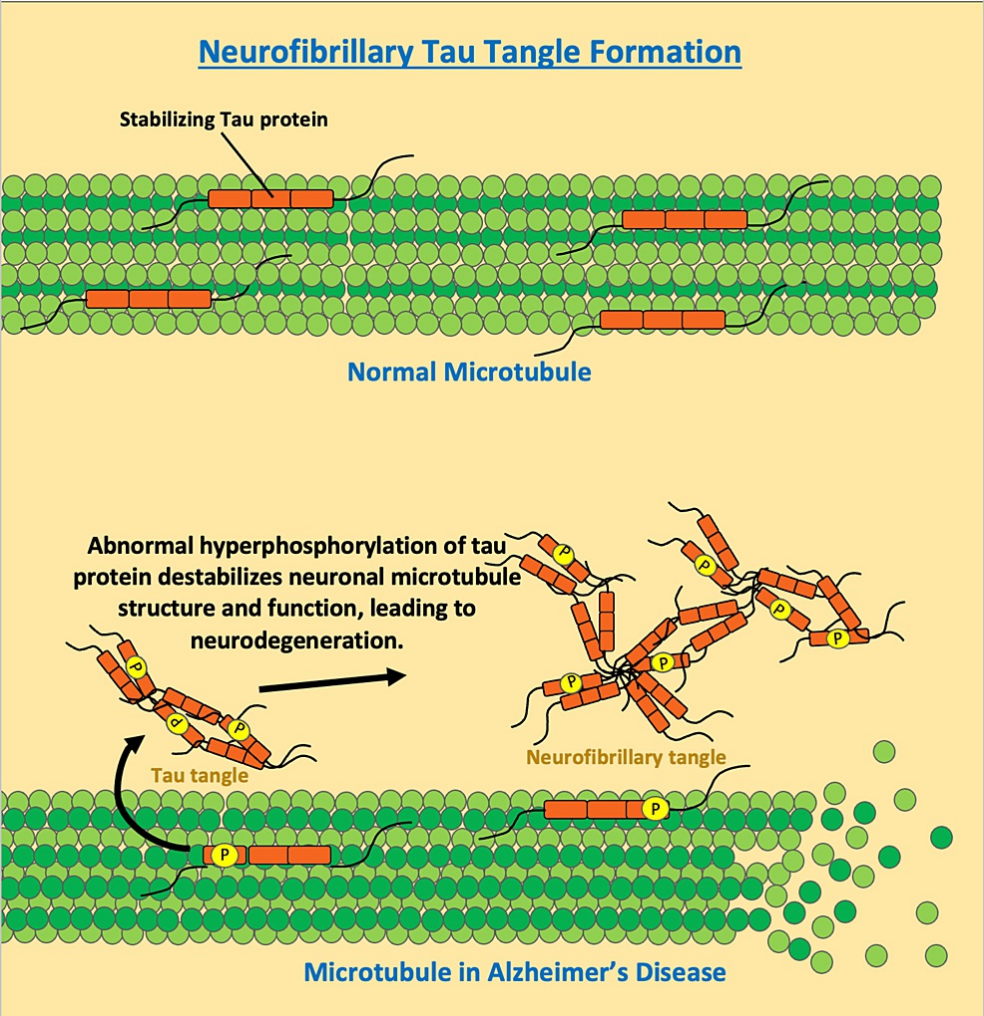
Neurofibrillary tau tangle formation Tau protein has a key role in stabilizing microtubule structure and function in neurons. The abnormal hyperphosphorylation of tau protein results in decreased affinity for microtubules and increased affinity for aggregation. As phosphorylated tau proteins aggregate, they form tau tangles, ultimately leading to the development of neurofibrillary tangles. The illustration was originally created by the authors.

This aggregation forms the hallmark amyloid-β plaques found in AD, which promote neurotoxicity by inducing oxidative stress and inflammation, triggering mitochondrial dysfunction, altering membrane permeability, and interfering with synaptic function resulting in neurodegeneration [5]. Neurofibrillary tangles consisting of hyperphosphorylated tau proteins are another histopathological hallmark of AD [5]. The tau protein is involved in the stabilization of microtubules, which are vital structures for the functioning of axonal transport and dendrites in neurons [5]. As illustrated by Figure 2, when tau protein is hyperphosphorylated it decreases its affinity for microtubules and increases its affinity for other tau proteins, forming neurofibrillary tangles [5].

Cognitive decline in AD progresses as the impaired functionality of tau proteins found in neurofibrillary tangles fails to augment neuronal connections and communication. Tau-dependent microtubule assembly, axonal trafficking, and dendrite structure formation are all key events needed for neurocognition [5].

Neuroinflammation is a key component in the pathology of AD. Chronic stimulation of cytokine-secreting microglia and astrocytes residing in the brain promotes an immune response that can potentially exacerbate amyloid-β deposition, tau hyperphosphorylation, and cerebral amyloid angiopathy [1]. Cerebral amyloid angiopathy is a term designated to characterize an accumulation of amyloid within or around cerebral blood vessels. Amyloid deposits around cerebral blood vessels alter the integrity of the blood-brain barrier and can lead to extravasations of plasma proteins, cerebral edema, and release of pro-inflammatory mediators [6]. Chronic neuroinflammation in AD is critical to the progression of the disease because it results in a positive feedback loop, where chronic neuroinflammation exacerbates the deposition of the histopathologic findings of AD, while at the same time, the deposition of these plaques and neurofibrillary tangles directly promote inflammation [1].

**Figure 3:**
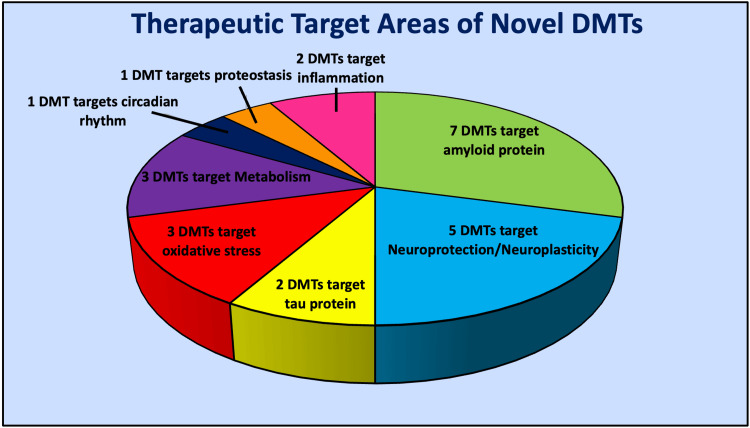
Therapeutic target diversity among novel disease-modifying treatments The image showing the therapeutic target diversity among novel disease modifying treatments (DMTs) recently approved by the United States Food and Drug Administration, or that are in phase 3 of clinical trials. The illustration was originally created by the authors.

There is a plethora of novel treatments for AD in development at several different phases in drug trials. One hundred and eighty-seven unique treatment agents for AD are undergoing trials as of January 1, 2023 [7]. Disease modification was by far the most common classification of drug agents undergoing testing in these trials as 79% of the agents targeted disease modification [7]. Disease-modifying treatment (DMT) is a label given to therapeutic agents whose purpose is to change the biology or slow the progression of AD [7]. It is reassuring that the major area of focus for drug development regarding novel treatments for AD is DMTs, as aducanumab and lecanemab are the only DMTs readily available today. This narrative review aims to investigate DMTs that have recently received U.S. Food and Drug Administration (FDA) approval in addition to DMTs in phase three of development that have the potential for approval in the near future. Figure 3 and Table 1 illustrate the diversity in therapeutic target areas of these novel DMTs.

**Table 1:** Novel drug agents and their therapeutic target areas

Therapeutic Target Area	Drug Agents
Amyloid accumulation	aducanumab, donanemab, gentenerumab , lecanemab, remternetug, solanezumab, valiltramiprosate
Neuroprotection/Neuroplasticity	AGB101 (levetiracetam), blarcamesine, fosgonimeton, simufilam , tertomotide
Oxidative Stress	hydralazine, hydrochloride, Icosapent ethyl, o mega-3
Metabolism	metformin, semaglutide, tricaprilin
Circadian Rhythm	piromelatine
Proteostasis	nilotinib
Inflammation	masitinib, NE3107 (b-androstenetriol derivative)
Tau accumulation	E2814 (monoclonal antibody), TRx0237 (methylthionium)

## Review

Novel drug agents’ and their therapeutic targets

Anti-amyloid

The largest and most common focus area of DMT agents is against the amyloid protein. Amyloid proteins aggregate to form the hallmark amyloid-β plaques found in AD, which promote neurotoxicity by inducing oxidative stress and inflammation, triggering mitochondrial dysfunction, altering membrane permeability, and interfering with synaptic function, ultimately leading to neurodegeneration [4]. Aducanumab is a novel monoclonal antibody that targets both amyloid-β plaques and amyloid oligomers in AD. The binding of aducanumab to amyloid-β plaques and oligomers in the brain helps stimulate and upregulate resident microglia in the clearance of said plaques [8]. It was the first disease-modifying drug approved by the FDA for mild AD in June of 2021 after showing promising results in reducing brain amyloid-β levels and slowing cognitive impairment in mild AD [8]. Lecanemab, only the second DMT to ever be approved by the FDA for AD in 2023, is a monoclonal antibody that selectively targets amyloid-β protofibrils [8]. Amyloid-β protofibrils are the toxic intermediates between misfolded amyloid monomers and amyloid fibril plaques in amyloid plaque formation [8]. Similar to aducanumab, lecanemab also showed a reduction in amyloid-β levels while slowing cognitive decline [8]. While both aducanumab and lecanemab showed a therapeutic response in patients with AD, lecanemab had a lower incidence of cerebral edema [8]. The FDA approved both of these agents on an accelerated pathway once they were shown to reduce the quantity of amyloid-$\beta$ plaques on amyloid Positron Emission Tomography scans in those with AD [7]. The FDA justified the approvals on the basis that a reduction in amyloid-β plaque formation would likely result in clinical benefit in those suffering from AD. The aforementioned anti-amyloid therapies unfortunately only help a limited segment of people suffering from AD, those with mild or early manifestations of the disease [7]. Gantenerumab and solanezumab are other monoclonal anti-amyloid therapies that resulted in disappointing therapeutic responses [8]. Donanemab and its analog remternetug are similar to other anti-amyloid therapies in this category; however, these drugs selectively target the pyroglutamate form of amyloid plaques [7]. Patients with mild AD treated with donanemab showed a reduction in both amyloid-β plaques and overall accumulation of tau proteins [9]. Valiltramiprosate, the prodrug of tramiprosate, falls within this class of medications and is being investigated as potentially the first oral DMT for AD [10,11]. However, the results of valiltramiprosate’s efficacy are still pending. 

Neuroplasticity and Neuroprotection

Neuroprotection and the induction of neuroplasticity were key focus areas for therapeutic benefit in patients with AD. Neuroprotection is crucial for slowing the damage AD does to the brain in early AD, while neuroplasticity is essential for forming and restructuring neuronal connections in brains affected by moderate to severe AD. Seventeen percent of phase 3 trials involved drug agents that targeted neuroprotection and neuroplasticity [7]. AGB101 is one of these agents. AGB101 is a low-dose formulation of the anticonvulsant medication, levetiracetam [12]. AGB101 exhibits its neuroprotective qualities by modulating synaptic vesicle glycoprotein 2A activity in the brain [7]. This modulation helps suppress neurotransmission and the potential excitotoxicity that comes with it. Blarcamesine, another drug in this class, acts through a different mechanism of action to promote neuroprotection and prevent excitotoxicity. Blarcamesine is a $\sigma$-1 receptor agonist and muscarinic-2 receptor antagonist [7]. Blarcamesine’s agonism at sigma-1 receptors prevents excitotoxicity by suppressing neuronal ion channel activity and transmission [13]. Fosgonimeton, hypothesized to promote neuroplasticity and the survival of hippocampal neurons, acts through hepatocyte growth factor receptor signaling [14]. Hepatocyte growth factor signaling has been implicated as a key component for neurite outgrowth and synaptogenesis, making it a promising drug target for restoring neural connections in patients with AD [14]. Simufilam is a small molecule DMT in this class that acts through the inhibition of Filamin-A, a scaffolding and regulator protein of the actin cytoskeleton [7]. In some experimental systems, Filamin-A was reported to be involved in the stabilization of the high-affinity interactional state of soluble amyloid-β-42 and the α-7 nicotinic acetylcholine receptor [15]. The interaction between amyloid-β-42 and the α-7 nicotinic acetylcholine receptor triggers tau protein phosphorylation and leads to synaptic dysfunction [15]. Filamin-A suppression by simufilam can therefore indirectly decrease abnormal tau protein phosphorylation in hopes of improving synaptic functioning in those with AD [7]. The last neuroprotective DMT agent in phase 3 trials is Tertomotide. Tertomotide is interesting because it was originally designed as an anticancer vaccine that activated the immune system for pancreatic malignancies but was later repurposed for AD [16]. The active component of this drug consists of a short peptide derived from the human telomerase reverse transcriptase (TERT) enzyme. Tertomotide’s derivation from the TERT enzyme and its ability to mimic TERT is proposed to be the reason for its antioxidant and antiapoptotic properties. Tertomotide demonstrates neuroprotection against amyloid-β plaques through its antioxidant and antiapoptotic properties, ability to stabilize mitochondria, and inhibition of inflammation-stimulated cellular proliferation [16].

Anti-tau

Monoclonal antibodies against tau proteins as a treatment for AD are also in development, although to a much lesser extent than the anti-amyloid therapies. The anti-tau monoclonal antibody, E2814, inhibits the aggregation of tau proteins by recognizing and binding the microtubule region tau proteins bind to. The microtubule-binding region that E2814 binds to is predominantly involved in the formation of neurofibrillary tau tangles [17]. Further aggregation of tau proteins into the growing neurofibrillary tangle is prevented by E2814’s binding to this microtubule region. Another DMT in this drug class, TRx0237, is in development and is the chemically reduced form of the most advanced tau protein aggregation inhibitor, methylthioninium [18]. TRx0237 existing as the reduced form of methylthioninium creates a less toxic and more bioavailable treatment option that targets tau protein aggregation [19]. TRx0237 showed promising results in reducing brain atrophy but was unable to slow cognitive decline and dysfunction in those with mild AD [20]. 

Oxidative Stress

Amyloid-β plaques cause oxidative stress to nearby lipid-containing cell membranes, leading to inflammation [21]. A class of DMTs in phase 3 of trials focuses on reducing this oxidative stress. Hydralazine hydrochloride is one of these. Hydralazine hydrochloride is able to reduce lipid oxidative damage through its hydrazide functional group and possesses antioxidant properties [21]. Hydralazine hydrochloride can prevent some of this oxidative stress by scavenging and removing the harmful products of lipid oxygenation. Hydralazine hydrochloride prevents lipid modifications of amyloid-β plaques like many hydrazides but is unique from other hydrazides in that it was shown to reduce amyloid-β misfolding [21]. Although hydralazine was capable of reducing amyloid-β misfolding, it was unable to reduce the aggregation of these misfolded proteins into their pathological plaques [21]. Another potential focus area for targeting oxidative stress in AD is through omega-3 fatty acids, or the purified form of one of these fatty acids, icosapent ethyl. Icosapent ethyl is a purified form of eicosapentaenoic acid and was previously confirmed to be cardioprotective [7]. The rationale for icosapent ethyl being a possible treatment option for AD is that eicosapentaenoic acid is capable of improving arterial and cerebrospinal blood flow [22]. An increase in cerebrospinal blood flow can improve the physiological functioning of the brain with various processes [22]. Icosapent ethyl and its effect on cerebrospinal blood flow may attenuate some of AD’s neurotoxic effects by improving the brain’s function in removing waste and toxins from the central nervous system (CNS). 

Metabolism

Late-onset AD has recently been characterized as a metabolic disorder, linked to an ineffective utilization of glucose by the brain [23]. Inefficient glucose utilization is correlated with disturbances in glucose and lipid metabolism, mitochondrial dysfunction, protein modification, and oxidative stress [23]. Furthermore, impaired glucose utilization and insulin resistance of the brain can enhance amyloid-β accumulation, and tau hyperphosphorylation, and impair hippocampal framework pathways [23]. Hyperglycemic drugs have recently garnered interest as a possible DMT for AD. The first-line agent for type 2 diabetes, metformin, is being investigated as a therapeutic agent for AD. This insulin sensitizer has shown both positive and negative contradictory findings in studies measuring age-related cognitive decline [23]. Despite the contradictory findings on metformin and age-related cognitive decline, metformin has proven benefits with its use in decreasing inflammation, improving memory, and accelerating neurogenesis [23]. Studies have recently demonstrated an association between metformin and neuroplasticity induction through metformin’s mechanism of enhancing synaptophysin, brain-derived neurotrophic factor, and sirtuin-1 levels in the brain [23]. Synaptophysin, brain-derived neurotrophic factor, and sirtuin-1 are essential markers of neuroplasticity [23]. Semaglutide is another hyperglycemic drug for type 2 diabetes that has garnered interest as a possible treatment for AD [7]. Semaglutide is a glucagon-like peptide-1 agonist that increases insulin sensitivity [7]. The use of semaglutide in clinical trials for AD is still lacking but has shown promising results with in-vitro studies. In-vitro studies involving a human-derived cell line damaged with amyloid-β showed the ability of semaglutide to protect against amyloid-β toxicity by increasing autophagy and decreasing apoptosis in the brain [24]. Tricaprilin is another DMT in this drug class that acts through a different mechanism of action. Brain hypometabolism is commonly associated with neurodegenerative disorders and tricaprilin is a treatment agent that can rectify this. Tricaprilin is a synthetic medium-chain triglyceride that can serve as an alternative energy source for the brain by serving as a precursor for ketogenesis [25]. The alternative energy source that tricaprilin-induced ketogenesis supplies the brain with has shown to have beneficial impacts on cognitive functioning in those with AD [25].

Inflammation

Chronic inflammation has been established as one of the main etiologies that can lead to the development of AD. This fact has brought about new research and development into DMTs that target inflammation for AD. A novel anti-inflammatory agent being investigated for AD is the tyrosine kinase inhibitor, masitinib [7]. Masitinib exhibits anti-inflammatory and neuroprotective properties by inhibiting mast cells and microglia [7]. When compared to a placebo, masitinib showed a significant improvement in both the cognitive subscale of the Alzheimer’s Disease Assessment Scale and the Activities of Daily Living Inventory Scale in the Alzheimer’s Disease Cooperative Study [26]. Another anti-inflammatory DMT is NE3107. NE3107 is a derivative of the anti-inflammatory and insulin-sensitizing adrenal sterol metabolite, β-androstenediol [27]. NE3107 suppresses inflammation by binding to extracellular signal-regulated kinase (ERK). The antagonism of ERK by NE3107 prevents the intracellular downstream activation of a nuclear transcription factor (NF-κB) and its resultant pro-inflammatory mediators [27]. 

Proteostasis

Dysregulated proteostasis and its resultant protein accumulation are hallmark characteristics in the pathology of AD. Some researchers are looking towards previous medications indicated for chronic myeloid leukemia as an alternative for AD. The DMT in this class is aimed at preventing the dysregulation and accumulation of abnormal proteins that occur in AD. Nilotinib is an anticancer drug that enhances autophagy in the CNS through its mechanism of inhibiting BCR-ABL tyrosine kinase [7]. The enhanced autophagy occurring in the CNS from nilotinib has been shown to lower both amyloid-$\beta$ plaque and hyperphosphorylated tau protein burden in those with AD [28]. Nilotinib also showed promising results in attenuating the loss of hippocampal brain volume that commonly happens in AD [28]. 

Circadian Rhythm

Circadian rhythm dysfunction is an extremely common finding in patients with AD, but its exact role in the disease’s pathogenesis is still unknown. A properly functioning central circadian rhythm is paramount for healthy brain functioning [29]. A dysfunctional circadian rhythm and its association with AD may be explained by the impaired function of the brain with processes such as removing toxins and waste products from the CNS. The accumulation of toxins and waste products in the CNS leads to chronic neuroinflammation. This neuroinflammation contributes to the development and progression of AD. Previous studies showed that amyloid-β levels exhibit diurnal oscillations that appear to be linked to the sleep-wake cycle [29]. Oscillating levels of amyloid-β may be explained by alterations in the brain’s neuronal activity and/or clearance of neuronal proteins that occur depending on the stage of the sleep-wake cycle. Patients with AD spend less time in the non-rapid eye movement (NREM) stage of sleep and exhibit reductions in slow-wave sleep activity [30]. Slow-wave sleep is an important stage of the sleep-wake cycle and has important roles in memory formation and glymphatic clearance. Reduced slow-wave sleep activity was implicated in impaired memory consolidation in those with AD [30]. Furthermore, sleep deprivation resulted in increased amyloid-β plaque deposition, while the promotion of adequate sleep via orexin inhibition resulted in a reduction in plaque accumulation [29]. The correlation between circadian rhythm, sleep disorders, and plaque burden led to researchers’ turn toward piromelatine as a possible treatment agent for AD [7]. Piromelatine is being explored as a treatment for AD due to its agonistic effects on melatonin and serotonin receptors, which may improve sleep quality [7,29]. Piromelatine may theoretically lower amyloid-β plaque burden and enhance memory consolidation in AD by restoring healthy sleep habits, although trials are still ongoing. Trials are still ongoing, but theoretically, agomelatine may reduce amyloid-β plaque accumulation and improve memory consolidation in AD by promoting healthier sleep habits.

## Conclusions

AD is a debilitating disease that is expected to become more prevalent as our population ages. Currently, only two DMTs are approved for AD treatment, but they are only beneficial to a limited segment of the AD population. The development of new drugs and therapies that target not just the symptoms, but also reverse or slow disease progression is imperative. Fortunately, there are several DMTs on the horizon that have the potential for FDA approval. Practicing clinicians should be cognizant of how these novel drugs differ in their therapeutic targets. Due to the complex and overarching pathoetiology of AD, a DMT with a particular therapeutic target may be more efficacious than other DMTs with different therapeutic targets, particularly if the therapeutic target of the DMT being administered aligns with the subpopulation’s most prominent pathological findings. However, future research comparing DMTs with different therapeutic targets and their efficacy in treating various populations of AD patients is needed for confirmation. The target areas of some DMTs that may soon be introduced clinically consist of therapies against amyloid and tau proteins, oxidative stress, inflammation, dysregulated proteostasis, dysregulated circadian rhythms, and dysregulated metabolism. Other DMTs that are on the horizon for FDA approval focus on neuroprotection or the promotion of neuroplasticity to slow the progression of AD.
